# Chronic Intraventricular Effusion of Chronic Hydrocephalus

**Published:** 1893-08-26

**Authors:** Bertram Rogers

**Affiliations:** Physician to the Bristol Children's Hospital


### CHRONIC INTRAVENTRICULAR EFFUSION OR CHRONIC HYDROCEPHALUS.

By Bertram Rogers, B.A., M.D. Oxon., Physician to the Bristol Children's Hospital.

There can be no difficulty in recognising a well marked case of chronic hydrocephalus, or as it is commonly known "water on the brain," but there are no symptoms peculiar to the disease, or which can be relied on as diagnostic, to assist us in forming a definite opinion on a suspected or doubtful case. The disease is one which is almost entirely confined to children. It begins in childhood, sometimes commencing during the period of interuterine existence, and producing under these circumstances such difficulty in parturition that puncture of the fœtal head has to be resorted to in order to save the maternal life, and it generally ends in childhood by the death of the patient from one cause or another. There are a few cases in which recovery takes place, and a still smaller number which reach adult life unrelieved. The term "chronic hydrocephalus" has been used rather laxly, so that under that head are included several conditions similar in condition but produced by very different and dissimilar causes. It would be preferable to speak of the disease as idiopathic intraventricular effusion, thereby admitting that in certain cases the ventricles of the brain are distended with fluid, from some cause, the exact nature of which we are at present ignorant. Such a definition at once places aside those cases in which effusion has taken place from recognised pathological conditions, such as those produced by pressure on the straight sinus by a tumour, and more rarely those in which an arachnoid hæmorrhage has taken place, or where there has been wasting of the cerebral hemispheres.

Idiopathic generally means that we cannot as yet assign a cause, and though in some cases of effusion there are signs of rickets or of syphilis, it is certainly not in the majority of cases that these diseases are present.

In a well marked case, as before stated, the recognition of the disease is simple enough. The enlarged head, with separation and thinning of the bones, the prominent bulging forehead, the depressed eyes, showing the sclerotic in the upper part, the apparent but not always real smallness of the face, and the frequent presence of some weakness or paralysis of the limbs, present a picture so peculiar and striking that even the beginner in medicine cannot fail to recognise the patient's condition. It is very doubtful whether a certain diagnosis can be made in the early stages of the disease; before the head begins to enlarge it is almost if not quite impossible; though one may have one's suspicions, one cannot speak with certainty. The size the head may reach varies much. It may be so slight that the diagnosis between a rickety enlargement and hydrocephalus is doubtful; and, on the other hand, the effusion may stretch the brain to such a degree of thinness that light may be transmitted through the head. Whatever the size of the head may be, the brain always shows the effect of the distention of its ventricles, the convolutions being flattened out till, in cases of great effusion, the cortex may be almost smooth. It appears that the brain in these cases is heavier than that of a healthy child of the same age, and this is probably due to an overgrowth of the neuroglia rather than a true hypertrophy of the brain.

The fluid, which in composition resembles very closely cerebro-spinal fluid, is contained in the lateral and third ventricles, but it sometimes bursts through the brain substance, though this phenomenon is rare. The quantities may be very large—as much as twenty-seven pints have been measured after death.

The course of the disease is, speaking generally, to an unfavourable conclusion, regardless of what treatment is adopted. But there are a certain number of severe cases—ones in which there has even been marked spastic rigidity of the legs—which improve, if not recover, often, however, leaving the patient in a more or less imbecile condition. It has been stated, by whom I have been unable to discover, that persons who have recovered from this disease exhibit more than usual mental capabilities, and the author of *Vanity Fair* has been instanced as an example, but it is probable that the sole foundation of this statement rests in the fact that that distinguished novelist had an unusually large head. In the writer's opinion it is more common than is generally supposed for a mild case to recover, and he believes that he has now three cases under his care which are undoubtedly recovering under general treatment. What the mental power of these children will be it will be interesting to observe, but it is hardly to be hoped that they will approach in intellect and literary power the gifted author mentioned above.

The treatment of this disease is far from satisfactory, and the usual statement to be found in text books on medicine is that drugs are incapable of causing absorption of the fluid, and so of curing the disease. Even in these cases in which amelioration of the symptoms takes place, it can hardly be claimed that the exhibition of any particular medicine has produced the effect. Of course, if there is any suspicion of a syphilitic taint being present in the patient, the appropriate drugs should be administered, and cases have been recorded of improvement following a course of mercury and iodides. In the same way rickets, with which the disease is sometimes associated, should be treated by the usual methods, but care should be exercised in an undoubted case of rickets not to mistake the enlarged skull of that disease with the distended head of hydrocephalus.

Local treatment has been adopted, the treatment being more empirical than theoretical. In this way pressure by bands of plaster applied all over the head has been employed, or more simply by a single band round the head. The greatest advocate of this plan of treatment is Dr. Dickinson, who recommended it in the case of young children with moderate enlargement of the head only, and by this means he claims to have cured a considerable number of cases. Great care has to be exercised in carrying out this line of treatment to avoid undue pressure on the already tightly stretched skin, or severe sloughing may take place, and lead to a fatal issue. Puncture of the head with an aspirating needle and the withdrawal of a small quantity of the fluid has been practised, and in some cases has been followed by some success. Antiseptic precautions will at the present time remove the fear of setting up inflammation within the head, but for all that the proceeding seems hardly justifiable to judge from the reports of the cases in which it has been employed. It has been suggested by Quincke, but as far as I can find there is no record of any cases so treated, to withdraw some fluid by inserting a needle between the third and fourth lumbar ventibræ, a space whence the spinal canal may be punctured without fear of wounding the cord. The suggestion is a fair one, but does not seem to promise success greater than puncture of the brain itself. Injection of fluids, such as iodine solutions, are useless, and yet are unattended with the harmful results that might be anticipated. Surgery has not so far lent its hand to cure this disease, but possibly some operation similar to that known by the name of craniectomy may be devised at a later date. Even in those cases where improvement is noted the practitioner will be disappointed if he hopes for a perfect cure, for the majority, if not all, of the patients, are mentally deficient to some degree or another.

